# Molecular Phylogenetic Analysis of *Paracoccidioides* Species Complex Present in Paracoccidioidomycosis Patient Tissue Samples

**DOI:** 10.3390/microorganisms11030562

**Published:** 2023-02-23

**Authors:** Luciana Bonome Zeminian de Oliveira, Amanda Manoel Della Coletta, Taiane Priscila Gardizani, Hans Garcia Garces, Eduardo Bagagli, Luciana Trilles, Ligia Vizeu Barrozo, Sílvio de Alencar Marques, Julio De Faveri, Luciane Alarcão Dias-Melicio

**Affiliations:** 1 Laboratory of Immunopathology and Infectious Agents—LIAI, UNIPEX—Experimental Research Unity—Sector 5, Medical School of Botucatu, São Paulo State University (UNESP), Botucatu 18618-687, SP, Brazil; 2 Department of Chemical and Biological Sciences, Institute of Biosciences, São Paulo State University (UNESP), Botucatu 18618-689, SP, Brazil; 3 Evandro Chagas National Institute of Infectious Disease, Oswaldo Cruz Foundation (FIOCRUZ), Rio de Janeiro 21040-900, RJ, Brazil; 4 Department of Geography, School of Philosophy, Literature and Human Sciences, University of São Paulo (USP), São Paulo 05508-080, SP, Brazil; 5 Department of Dermatology, Medical School of Botucatu, São Paulo State University (UNESP), Botucatu 18618-687, SP, Brazil; 6 Department of Pathology, Medical School of Botucatu, São Paulo State University (UNESP), Botucatu 18618-687, SP, Brazil

**Keywords:** *Paracoccidioides* species complex, *Paracoccidioides brasiliensis*, molecular phylogenetic analysis, paracoccidiodomycosis, molecular biology

## Abstract

Paracoccidioidomycosis (PCM) is the main and most prevalent systemic mycosis in Latin America, that until recently, it was believed to be caused only by *Paracoccidioides brasiliensis* (*P. brasiliensis*). In 2006, researchers described three cryptic species: S1, PS2, PS3, and later, another one, PS4. In 2009, *Paracoccidioides lutzii* (Pb01-like) was described, and in 2017, a new nomenclature was proposed for the different agents: *P. brasiliensis* (S1), *P. americana* (PS2), *P. restrepiensis* (PS3), and *P. venezuelensis* (PS4). These species are not uniformly distributed throughout Latin America and, knowing that more than one cryptic species could coexist in some regions, we aimed to identify those species in patients’ biopsy samples for a better understanding of the distribution and occurrence of these recently described species in Botucatu region. The Hospital of Medical School of Botucatu—UNESP, which is a PCM study pole, is located in São Paulo State mid-west region and is classified as a PCM endemic area. Genotyping analyses of clinical specimens from these patients that have been diagnosed and treated in our Hospital could favor a possible correlation between genetic groups and mycological and clinical characteristics. For this, molecular techniques to differentiate *Paracoccidioides* species in these biopsies, such as DNA extraction, PCR, and sequencing of three target genes (ITS, CHS2, and ARF) were conducted. All the sequences were analyzed at BLAST to testify the presence of *P. brasiliensis*. The phylogenetic trees were constructed using Mega 7.0 software and showed that 100% of our positive samples were from S1 cryptic species, therefore *P. brasiliensis*. This is important data, demonstrating the predominance of this species in the São Paulo State region.

## 1. Introduction

Paracoccidioidomycosis (PCM) is a systemic granulomatous disease affecting mainly the lungs, being restricted to them or being disseminated to any other organs through hematogenous lymph circulation, such as the bone, liver, spleen, and central nervous system, resulting in several different and severe clinical manifestations, characterizing the acute/subacute or chronic forms [1]. PCM is an important neglected tropical disease where most of the cases occur in Brazil, Colombia, Venezuela, and Argentina. Cases described in other countries outside Latin America occurred in patients that previously lived in endemic areas [2,3,4,5,6].

Mycotic infections are on the rise worldwide, and the emergence and re-emergence of fungal pathogens are related to climate change, deforestation, agricultural practices, biodiversity loss, human occupation, and uncontrolled use of immunosuppressive drugs [7].

The etiologic agents of PCM are the fungi encompassed in the genus *Paracocidioides*. They are thermodimorphic microorganisms that grow as yeast in vivo, in host tissues or cultures at 37 °C in enriched culture media. They are also presented as mycelium at room temperature ranging from 4 to 28 °C [8,9,10,11].

*Paracoccidioides brasiliensis* was believed to be the sole etiologic agent causing PCM, but studies of genomics and phylogeny pointed out that there are more than one species able to cause this mycosis. In 2006, three cryptic species (S1, PS2, and PS3) were described and, in 2009, *Paracoccidioides lutzii* [8,12]. Thereafter, a fourth cryptic species of *P. brasiliensis*, PS4, was described [9]. Recently, it was proposed to elevate the four phylogenetic species to formally describe taxonomic species as follows: S1 (*P. brasiliensis*), PS2 (*P. americana*), PS3 (*P. restrepiensis*), PS4 (*P. venezuelensis*) [8,9,10,11].

These species are not uniformly distributed in Latin America, where some are more prominent in determined regions than in others [8,9,12,13,14,15,16,17,18,19]. Phylogenetic species S1a and S1b are predominant in southeastern and southern Brazil, Argentina, and Paraguay. The PS2 species has a sporadic distribution and is less frequently found in cases reported thus far in southeast Brazil and Venezuela. The PS3 and PS4 species are exclusively from Colombia and Venezuela, respectively, and *P. lutzii* is predominantly distributed in the Amazon and central west regions of Brazil and Ecuador [2,6,8,9,10,11,12,13,14,15,16,17,18,19,20,21,22,23]. 

Defining species boundaries is a challenge in fungi. The most commonly used parameters are morphological characteristics and, sometimes, the competence to produce fertile offspring. In fungi, these characteristics that distinguish species are not simple to find and the species may become genetically isolated before developing such characteristics [13].

Identification and classification of eukaryotes increasingly depend on DNA sequences of standardized genetic markers, a concept known as DNA barcoding [24,25,26,27,28]. The modern concept of fungal DNA barcoding does not differ substantially from approaches proposed more than two decades ago. Sanger DNA sequencing of nuclear rDNA domains remains the most widely accepted approach in molecular mycology to classify and identify unknown fungal specimens or cultures. The first comprehensive database of fungal DNA barcodes was established for yeasts and revolutionized the approach to species recognition in that group [29].

Matute et al. [12] described the speciation of *Paracoccidioides* spp. using *multilocus* genealogy analyses, demonstrating that its genetic variability could be more than just a polymorphism characterizing the cryptic species. This means that their divergence was not explained by the known phenotypic profiles that could not distinguish the species. Five genes were sequenced to make this distinction: CHS2 (chitin synthase), FKS (β-glucan synthase), α-tubulin, ARF (adenyl ribosylation factor), and GP43 (immunodominant glycoprotein). Even though there is no agreement if the cryptic species are just geographic variants of the same species or distinct species, they are reproductively isolated [8,12,13]. Since reproductive and genetic isolations are the first steps in species divergence, it could lead to morphological and physiological differences with important consequences for the diagnostics and treatment of PCM [13]. 

With the knowledge that more than one cryptic species coexists in some regions, it was extremely valuable to analyze the presence of different *Paracoccidioides* species in patients’ biopsy samples, for a better understanding of the distribution and occurrence of these species in our endemic area and, also, a possible correlation between the genetic group and mycological and clinical manifestations [30]. The Medical School of Botucatu (FMB) of São Paulo State University (UNESP) is a public institution, considered the mycosis study pole in Botucatu, a city located in the mid-west region of the São Paulo State in Brazil, which is classified as a hyperendemic area of this important neglected mycosis. Moreover, given the formalin-fixed paraffin-embedded (FFPE) tissue samples collection of PCM patients treated in the Hospital of UNESP Medical School of Botucatu, this study sought to apply molecular techniques to differentiate these *Paracoccidioides* species in stored patients’ biopsies, contributing for a better understanding of species distribution in this important region of São Paulo State.

## 2. Materials and Methods

### 2.1. Study Setting, Data, and Sample Collection

The tissue samples used in this study were preserved with paraffin and stored at the Pathology Department of UNESP Medical School of Botucatu and are from 177 patients with confirmed PCM by histological analysis during the period from 2004 to 2014.

This research was conducted after approval by the Research Ethics Committee of UNESP Medical School of Botucatu (CAAE: 31053514.1.0000.5411). The Research Ethics Committee exempted the use of consent forms for our patients given the retrospective nature of our study.

As a control group, the study included 05 (five) fresh tissue samples collected from patients admitted to the UNESP Medical School of Botucatu hospital in 2017. All these patients agreed to participate by signing a consent form and had paracoccidioidomycosis diagnosis by serological and histopathological exams. Clinical history and socio-demographic profile were collected from patients’ records.

### 2.2. DNA Extraction

The samples were cut in a microtome and 3 portions of 8 µm thickness that showed a large fragment of tissue were used. Five to seven portions of 8 µm thickness were used for the samples containing smaller fragments of tissue. The blades were changed for each paraffin block to avoid contamination. 

Once in microcentrifuge tubes, the portions were submitted to xylol and alcohol washes until the paraffin melted completely. From this point, the DNA extraction of the 177 samples was conducted using commercially available kits, QIAmp DNA Mini Kit and QIAmp FFPE DNA Tissue Kit (Qiagen, Hilden, Germany), according to the manufacturer’s instructions. The extracted DNA was quantified by spectrophotometry (Epoch Life Sciences, Inc., Missouri City, TX, USA).

To evaluate the effect of paraffin in tissue samples, we extracted DNA from 05 fresh tissue samples that were collected in a biopsy procedure and frozen until use. The DNA extraction was conducted using a commercially available kit, QIAmp DNA Mini Kit (Qiagen, Hilden, Germany), according to the manufacturer’s instructions.

### 2.3. PCR Amplification of Target Loci ITS, ARF, CHS2, and Agarose Gel Electrophoresis

Nested PCR protocol for the ITS was used to certify the presence of *P. brasiliensis*. The genes’ ADP-ribosylation factor (ARF) and chitin synthase 2 (CHS2) were amplified according to Matute et al. [12]. The primers used in this technique are displayed in Table 1.

The master mix for the first round of the nested-PCR for ITS consisted of 1× Platinum Taq DNA Polymerase 10× buffer (Invitrogen), 1.5 mM of MgCl_2_ (Invitrogen), 0.2 mM of deoxynucleotides (Invitrogen), 0.6 µM of each primer (ITS4 e ITS5—Table 1) (Invitrogen), 1 U of Platinum Taq DNA Polymerase (Invitrogen), and 4 µL of DNA on final volume of 25 µL. It was incubated at 94 °C for 5 min, 35 cycles of 95 °C for 1 min, 53 °C for 1 min, 72 °C for 1 min, and a final extension at 72 °C for 5 min.

For the second (nested) PCR, the mixture was the same only using the inner pair of primers (PBITS-E and PBITS-T—Table 1) and 4 µL of the first PCR on final volume of 25 µL and changing the annealing temperature to 54 °C.

The master mix for the PCR for the ARF gene consisted of 1× Platinum Taq DNA Polymerase 10× buffer (Invitrogen), 1.5 mM of MgCl_2_ (Invitrogen), 0.2 mM of deoxynucleotides (Invitrogen), 0,6 µM of each primer (ARF-FWD e ARF-REV—Table 1) (Invitrogen), 1 U of Platinum Taq DNA Polymerase (Invitrogen), and 4 µL of DNA on final volume of 25 µL. It was incubated at 94 °C for 5 min, 35 cycles of 95 °C for 1 min, 57 °C for 1 min, 72 °C for 1 min, and a final extension at 72 °C for 5 min.

The master mix for the PCR for the CHS2 gene consisted of 1× Platinum Taq DNA Polymerase 10× buffer (Invitrogen), 1.5 mM of MgCl_2_ (Invitrogen), 0.2 mM of deoxynucleotides (Invitrogen), 0,6 µM of each primer (CHS2 E2–4 Fwd e CHS2 E2–4 Rev—Table 1) (Invitrogen), 1 U of Platinum Taq DNA Polymerase (Invitrogen), and 4 µL of DNA on final volume of 25 µL. It was incubated at 94 °C for 5 min, 35 cycles of 95 °C for 1 min, 55 °C for 1 min, 72 °C for 1 min, and a final extension at 72 °C for 5 min.

The revelation of the PCR assays was performed using agarose gel electrophoresis with agarose 2% gel electrophoresis using 1× TBE buffer and the conditions were 90 volts for 45 min.

### 2.4. Purification and Sequencing of Target Loci ITS, ARF, and CHS2

The PCR product from the extracted DNA with FFPE kit was cut from the 1.5% agarose gel, purified with Sequencing Clean-Up kit or, in the case of the fresh tissue samples, ExoProStar 1-Step Enzymatic PCR (GE Healthcare, Little Chalfont, UK) according to manufacturer’s instructions.

For the sequencing reaction, the master mix used consisted of 1.5 µL of Big Dye Terminator v3.1 5× Buffer, 1 µL of Big Dye Terminator v3.1, 1 µL of primer (Table 1), 4 µL of purified product, and 2.5 µL of sterile water. The reaction is performed separately for each primer (forward and reverse). The conditions were 95 °C for 1 min, 40 cycles 95 °C for 10 s, 50 °C for 10 s (temperature decrease 1 °C per second), and 60 °C for 4 min.

The DNA precipitation protocol used was to add 50 µL of ethanol, 2 µL of sodium acetate (3 M), and 2 µL of EDTA (125 mM) in each well. The plate is incubated for 15 min without light incidence and centrifuged for 45 min at 2000× *g* at 4 °C. After that, invert the plate and briefly centrifuge at 200× *g* with the plate still inverted. Then, add 70 µL of 70% ethanol in each well and centrifuge for 15 min at 2000× *g* at 4 °C. Again, invert the plate and briefly centrifuge at 200× *g* with the plate still inverted. The ethanol excess was dried in a thermocycler with the lid open at 90 °C for 2 min.

For the denaturation process, 10 µL of formamide was added into each well and incubated in a thermocycler for 3 min at 95 °C. Put the plate on ice right after for 2 min to perform the thermal shock. The plates were read in a 3500 Series Genetic Analyzer (Thermo Fisher Scientific, Waltham, MA, USA).

### 2.5. Sequences Analysis 

The ITS gene sequences were compared with sequences deposited at GenBank to testify to the presence of *Paracoccidioides* sp. Then, the ITS, ARF, and CHS2 gene sequences were used to construct the phylogenetic trees using MEGA 7.0.

For the ITS, CHS2, and ARF gene phylogenetic trees, the evolutionary history was inferred by using the Maximum Likelihood method based on the Tamura 3-parameter model and 1000 copies of bootstrap. In the phylogenetic trees, our sample sequences were compared with sequences from S1, PS2, PS3, and Pb01-like deposited by Matute et al. [12].

## 3. Results

### 3.1. DNA Extraction and PCR Amplification of Target Loci ITS, ARF, and CHS2, and Agarose Gel Electrophoresis

From 177 samples of patients with confirmed PCM, QIAmp DNA Mini Kit produced 24 positive samples and the QIAmp FFPE DNA Tissue Kit produced 44 positive samples. So, the last one was used for analysis, which is specific to FFPE tissue samples. This technique also uses a silica column-based method with an extra incubation period in high temperatures to try to minimize the damages caused by the fixation with formalin-breaking cross linkages; 44 positive samples were obtained, 20 more than that already amplified, showing a better outcome of positive samples, although at a higher cost. It was also chosen to perform a purification by cutting bands from agarose gel. The process is more time-consuming than the enzymatic or column-based techniques but showed more efficiency in our samples. For the genes’ PCR amplification, the figures show the ITS gene, identified as a 450 base pairs band (Figure S1), the ARF gene as a 407 base pairs band (Figure S2), and the CHS2 gene as a 549 base pairs band (Figure S3) (Supplementary Material). All the fresh tissue samples tested (*n* = 5) were positive in both tests, totaling 49 identified samples in this study. 

### 3.2. Clinical and Socio-Demographic Data 

Clinical and socio-demographic data are also shown for 49 patients with paracoccidioidomycosis (Table 2). The results showed that the chronic type of PCM is more common in our region predominating in 77.55% of the cases. The disease was more frequently presented in males (86.84%), white individuals (81.58%), smokers (89.47%), and alcoholics (63.16%), with a median age superior to 50 years old. In the acute type, the data are more uniform between genders, although predominant in white individuals (81.82%), non-smokers (81.82%), and non-alcoholics (100%) (Table 2).

**Figure 1:**
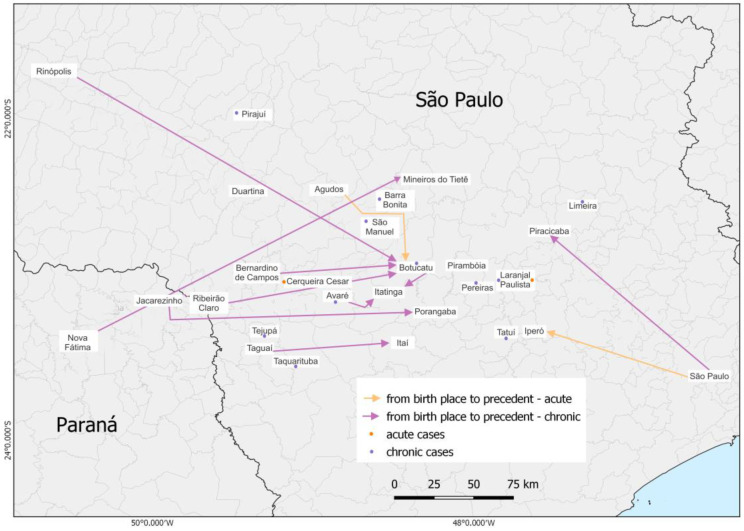
Map demonstrating the distribution of the 49 patients’ birthplace, provenance, and clinical form (11 acute cases and 38 chronic cases).

The distribution of patients according to birthplace and provenance as well as the clinical form of the disease was placed on a map (Figure 1), which shows most patients with the chronic form, including patients from another state (Paraná). It was identified that most of our patients are residents of the São Paulo State with a few cases from Paraná. The analysis of the area showed that where the patients lived, the place where they came from at the moment of the PCM diagnosis and treatment start, was predominantly concentrated in municipalities at São Paulo State central west region, around the city of Botucatu, corroborating the data published in 2021 [32].

### 3.3. Purification and Sequences Analysis: ITS, CHS2, and ARF Target Loci

After the sequencing of our target genes and ITS, sequences were compared with sequences in the GenBank database testifying the presence of *P. brasiliensis*. 

**Figure 2:**
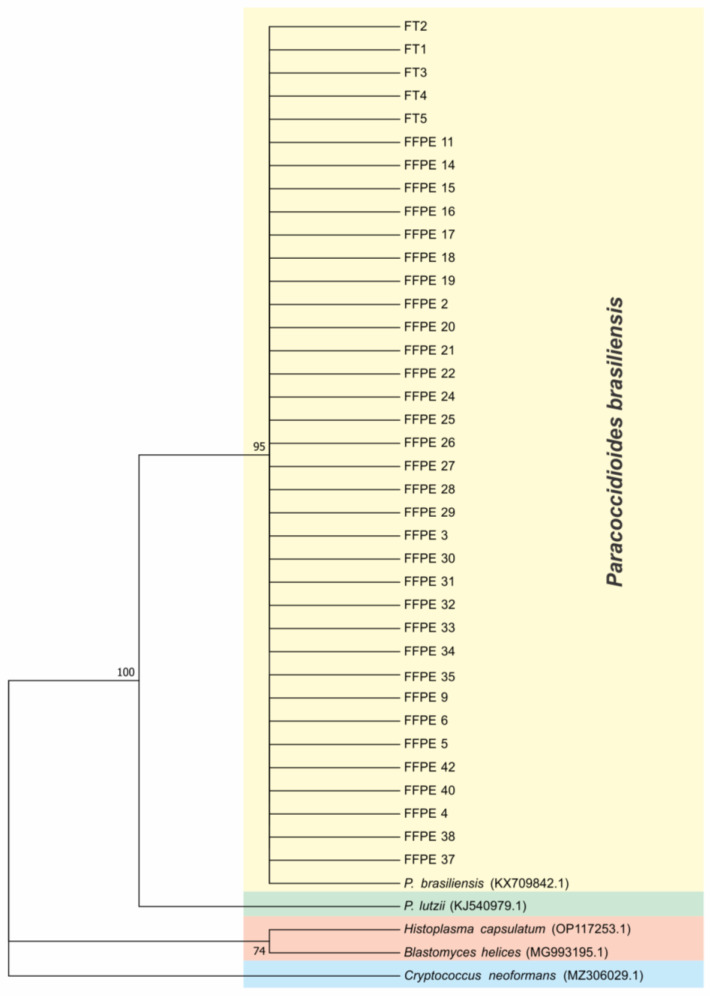
ITS *loci* phylogenetic tree using Molecular Phylogenetic analysis by Maximum Likelihood method based on the Tamura–Nei model. The tree with the highest log likelihood (−702.78) is shown. The percentage of trees in which the associated taxa are clustered together is shown next to the branches. Initial tree(s) for the heuristic search were obtained automatically by applying Neighbor-Join and BioNJ algorithms to a matrix of pairwise distances estimated using the Maximum Composite Likelihood (MCL) approach, and then selecting the topology with superior log likelihood value. The tree is drawn to scale, with branch lengths measured in the number of substitutions per site. The analysis involved 42 nucleotide sequences. All positions containing gaps and missing data were eliminated. There were a total of 263 positions in the final dataset. Evolutionary analyses were conducted in MEGA7.

**Figure 3:**
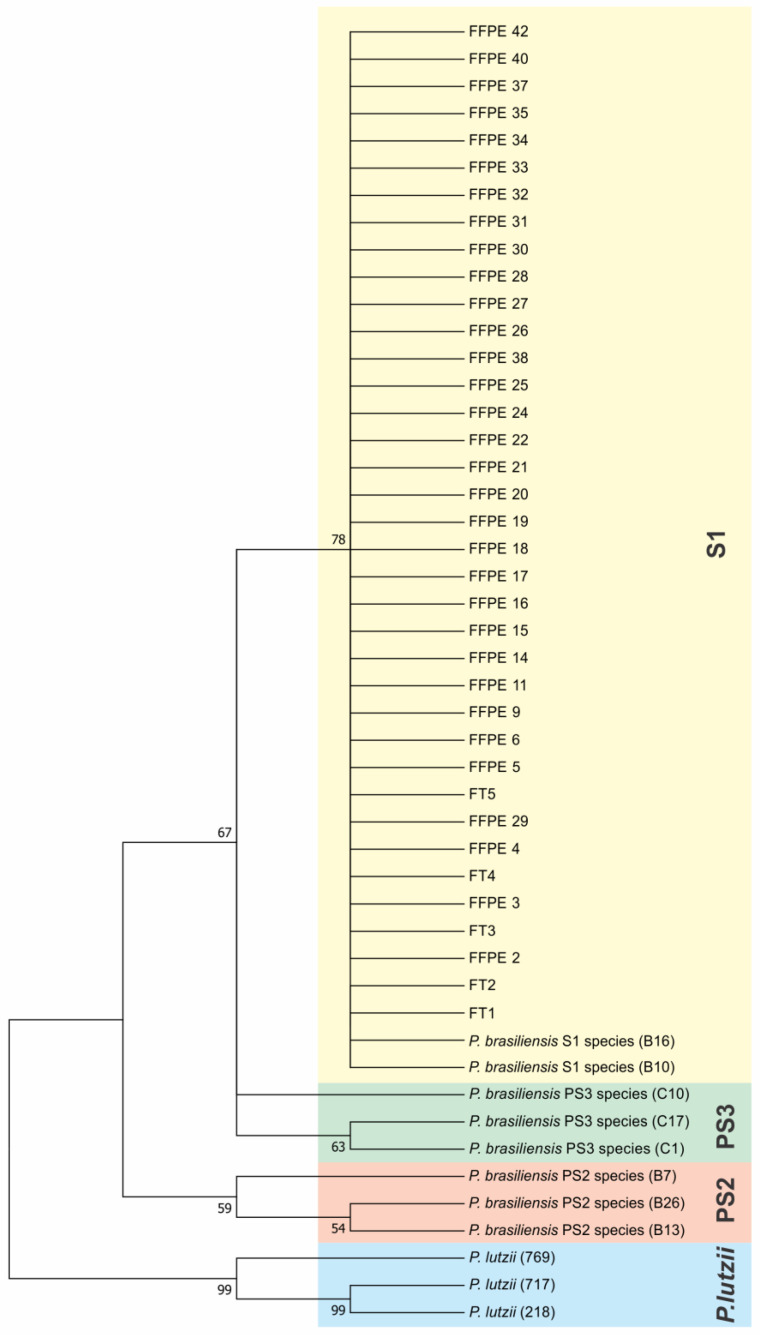
Concatenated ARF and CHS2 genes’ phylogenetic tree using the Maximum Likelihood method based on the Tamura–Nei model. The tree with the highest log likelihood (−1354.77) is shown. The percentage of trees in which the associated taxa are clustered together is shown next to the branches. Initial tree(s) for the heuristic search were obtained automatically by applying Neighbor-Join and BioNJ algorithms to a matrix of pairwise distances estimated using the Maximum Composite Likelihood (MCL) approach, and then selecting the topology with superior log likelihood value. The tree is drawn to scale, with branch lengths measured in the number of substitutions per site. The analysis involved 48 nucleotide sequences. All positions containing gaps and missing data were eliminated. There were a total of 776 positions in the final dataset. Evolutionary analyses were conducted in MEGA7.

Phylogenetic trees were constructed with the sequences of ITS (Figure 2). To compare and identify our isolates, we constructed a phylogenetic tree with other fungal ITS sequences such as *Histoplasma capsulatum* (PU20Z14095-direct submission), *Blastomyces helices* (MG993195.1) [33], *Cryptococcus neoformans* (MZ306029.1-direct submission), *Paracoccidioides lutzii* (KJ540979.1-direct submission), and *Paracoccidioides brasiliensis* (KX709842.1-direct submission). We constructed a concatenated phylogenic tree with both ARF and CHS2 genes (Figure 3) using the same methods, showing that 100% of our samples belonged to the S1 cryptic species. To certify our findings, we compared with samples from S1, PS2, PS3, and *P. lutzii* species described by other authors [8,12]. The positive fresh samples collected from PCM patients’ lesions are also of the S1 species (Figure 2 and Figure 3). The sequences and blast of ITS, CHS2, and ARF target genes are shown in Figures S4–S6. The GenBank sequence accession numbers from our samples are shown in Table S1 and the accession numbers from the samples used in the ARF and CHS2 tree are shown in Table S2 (Supplementary Materials).

## 4. Discussion

PCM is considered a neglected disease, emphasizing a vicious circle of poor healthcare conditions and poverty [4]. This illness has a profound socioeconomic impact since it affects mostly rural male workers, 30–50 years old, who are the main providers, hindering their ability to work [34]. Our clinical and socio-demographic data corroborate with the known literature regarding PCM [4,32], showing that chronic form was more frequently presented in males, white individuals, smokers, and alcoholics, with a median of age superior to 50 years old, as already described; while acute form affects both genders, white individuals, non-alcoholics, non-smokers, and of younger age [32,35,36]. 

Knowing that S1 (*P. brasiliensis*) normally occurs in southeastern and southern Brazil, and PS2 (*P. americana*) could be less frequently identified in southeast Brazil [4,6,7,8,9,10,11,12], it became interesting to evaluate which species infected our patients, as our hospital is located in the mid-west region of the São Paulo State in Brazil, classified as an important hyperendemic area of the disease. Thus, an important issue of our study was also to identify the *Paracoccidioides* spp. by genotyping analyses of clinical specimens from the patients treated at The Medical School of Botucatu (FMB) of São Paulo State University (UNESP). Molecular techniques can detect biomarkers such as DNA, RNA, and gene products from etiologic agents of the most varied diseases. Considering *Paracoccidioides* spp., several molecular techniques are used for the detection of fungi from soil samples in urban and rural environments and aerosols, helping and making it easier for ecological studies and geographical tracking [37]. However, the same may not be valid for clinical routine, since many variables must be considered. Additionally, the taxonomy of PCM agents went through several changes since the discovery of the disease at the beginning of the last century, stimulated by the massive morphological diversity [38,39] and differences in the genetic features of some isolates. Such characterizations have intensified in recent years by introducing molecular methods for the genetic differentiation of fungi, such as multilocus sequencing analysis [8,11,12,13] or whole-genome sequencing analysis [37,40,41]. Sequencing followed by phylogenetic analysis is considered the gold standard for the species identification of *Paracoccidioides*, but it may present limitations depending on the locus used [42]. Nuclear coding genes, including chitin synthase (CHS2), ADP-ribosylation factor (ARF), and an internal transcribed spacer (ITS) were used to identify the species on our samples. This scheme could separate *P. brasiliensis* complex members into distinct groups, allowing the identification of the species [42].

Usually, the lesion of the patients is biopsied for histopathological analysis, and during the tissue fixation process, formalin is used to stabilize tissue architecture and morphology, causing a consolidation that could endure staining and prevents decomposition. However, this procedure could induce serious harmful effects on nucleic acids, and converting the task of extracting good-quality DNA from these samples is a big challenge [43,44,45,46,47,48,49,50,51,52,53,54,55,56,57,58]. This methodology presented itself as our biggest challenge during the development of this study. 

The outcomes and the difficulties during PCR amplification using these kinds of samples were corroborated by literature [55,56,59,60,61,62]. Nevertheless, in this analysis, our samples provided a satisfying quantity of DNA allowing us to identify our target genes. Another characteristic of the extracted DNA from our FFPE samples showed fungal DNA in samples dated from 2004 and 2006, demonstrating that the positivity is not dependent on the period of storage. Several works described in the literature demonstrate that using FFPE tissues that were fixated with formalin has low positivity rates and consequent difficulty in PCR amplification and sequencing among other molecular techniques [63]. Smaller-sized fragments up until 550 base pairs were easier to successfully amplify. To demonstrate that the problems were related to the sample fixation process, the same techniques were performed in fresh tissue samples collected from new patients that were diagnosed during the year 2017. From January to October, 5 fresh tissue samples were received, and all were positive for *Paracoccidioides*. These data clearly show that our obstacle was indeed the fixation process.

Outbreaks of PCM were never observed and fungal recovery from the environmental form was shown to be extremely difficult to acquire. However, a cluster of acute/subacute cases of PCM, associated with a climatic anomaly, was observed in the endemic area of Botucatu [64], and a similar situation was also observed in Rio de Janeiro State, after a highway construction [65]. Although, more sensitive molecular techniques were able to find the pathogen in the soil and aerosols [14,34,66,67,68,69]. Given the *Paracoccidioides* sp. species distribution, an updated endemic map was recently published [1,2,3,4]. The map specifically shows that our endemic area has a prevalence for the S1 (*P. brasiliensis*) species. With this information, our data corroborate the latest published information validating our efforts, since the S1 (*P. brasiliensis*) species in all samples tested was identified. Additionally, according to the literature, the Botucatu region is a renowned treatment center for PCM which could explain the migration and possibly, the place of contamination, given that our region is extremely endemic for the *Paracoccidioides* sp.

There is no scientific evidence of whether different species cause different clinical manifestations. Therefore, our results could also answer this issue. However, among our patients, there were cases of juvenile form as well as chronic type. This suggests that regardless of the species, both clinical forms could manifest. It was also aimed to correlate the sociodemographic and clinical features with the mycological data, but the genotyping of our samples showed that all fungi present in samples were S1 species, preventing a more profound epidemiological and clinical correlation. These results could indicate that the immune response capacity of each individual would be responsible for establishing the clinical form of the disease, being more important than the infecting species.

Although our results did not demonstrate, among our group of patients tested, any other species such as PS2 (*P. americana*), its presence was already detected in low frequency in the Botucatu endemic area, both in human (BT84 isolate) and in armadillo isolates (T10B1 and T18LM3–5 isolates) [19,70,71] as well as in another São Paulo State region (Ribeirão Preto), also in a very low rate [67]. Thus, as originally a larger number of samples were gathered in our study, but only 49 samples were positive, we cannot assure that among the other samples, there was not any patient infected with PS2 species (*P. americana*).

Therefore, in this study, significant results were obtained regarding the prevalence of species in the Botucatu region, once only the S1 cryptic species (*P. brasiliensis*) was identified. It was also shown that working with fresh tissue collected from PCM lesions is much more effective for molecular analysis than FFPE samples, which resulted in 100% of positivity. This analysis enabled the achievement of a more detailed epidemiological background of the affected individuals in the Botucatu endemic region. 

## Figures and Tables

**Table 1:** Primers used in PCR amplification for our target *loci*.

**Gene**	**Primer**	**Sequence (5′-3′)**	**Amplicon**	**Reference**
Nested ITS first round	ITS4	TCCTCCGCTTATTGATATGC	634 bp	[31]
	ITS5	GGAAGTAAAAGTCGTAACAAGG		
Nested ITS second round	PbITS-E	GAGCTTTGACGTCTGAGACC	450 bp	[19]
	Pb-ITST	GTATCCCTACCTGATCCGAG		
ARF	ARF-FWD	TCTCATGGTTGGCCTCGATGCTGCC	407 bp	[12]
	ARF-REV	GAGCCTCGACGACACGGTCACGATC		
CHS2	CHS2 E2–4 Fwd	CTTAACGGTGCCTTCTTTGCGG	549 bp	[12]
	CHS2 E2–4 Rev	GTGAAAGTATTGTTGCCCAGCG		

**Table 2:** Sociodemographic characteristics and relevant clinical parameters of patients treated in The Medical School of Botucatu (FMB) of São Paulo State University (UNESP), Botucatu, from 2004 through 2014 and 2017.

Characteristics		Acute (*n* = 11)	Chronic (*n* = 38)	*p*-Value
Patient’ age (years) *		27.2(8–45)	53.8 (27–81)	-
Gender **	Male	54.55% (6/11)	86.84% (33/38)	0.0327
	Female	45.45% (5/11)	13.16% (5/38)	0.0327
Ethnicity **	White	81.82% (9/11)	81.58% (31/38)	NS (*p* < 0.05)
	Non-white	18.18% (2/11)	18.42% (7/38)	NS (*p* < 0.05)
Smokers **	Smoker	18.18% (2/11)	89.47% (34/38)	<0.0001
	Non-smoker	81.82% (9/11)	10.53% (4/38)	<0.0001
Alcoholics **	Alcoholic	0% (0/11)	63.16% (24/38)	0.0002
	Non-alcoholic	100% (11/11)	36.84% (14/38)	0.0002

* Values expressed as median (min-max); ** Values expressed in percentage.

## Data Availability

All relevant data are within the paper and its Supplementary Materials.

## Supplementary Materials

The following supporting information can be downloaded at: https://www.mdpi.com/article/10.3390/microorganisms11030562/s1.
