# Supplementary material for: Molecular Phylogenetic Analysis of Paracoccidioides Species Complex Present in Paracoccidioidomycosis Patient Tissue Samples
Source: Microorganisms. 2023 Feb 23;11(3):562. doi: 10.3390/microorganisms11030562 (PMC10055015; doi:10.3390/microorganisms11030562)
Supplement: Supplementary file 1 [file microorganisms-11-00562-s001.zip › microorganisms-2100971-supplementary.pdf]

microorganisms

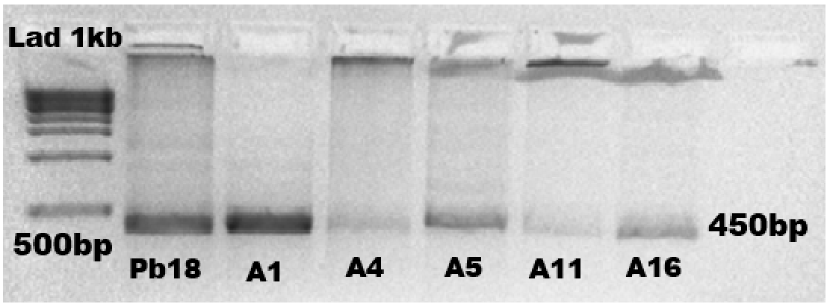

Figure S1. Amplification of the ITS gene (450 base pairs) in 2% agarose gel photographed using GE Image Quant LAS 4000 (GE Healthcare, Little Chalfont, United Kingdom).

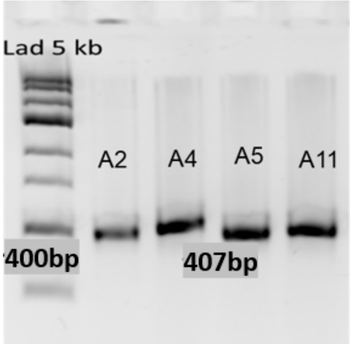

Figure S2. Amplification of the ARF gene (407 base pairs) in 2% agarose gel photographed using GE Image Quant LAS 4000 (GE Healthcare, Little Chalfont, United Kingdom).

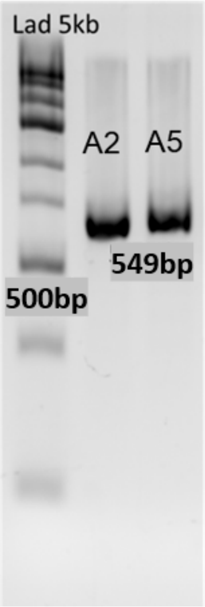

Figure S3. Amplification of the CHS2 gene (549 base pairs) in 2% agarose gel photographed using GE Image Quant LAS 4000 (GE Healthcare, Little Chalfont, United Kingdom).

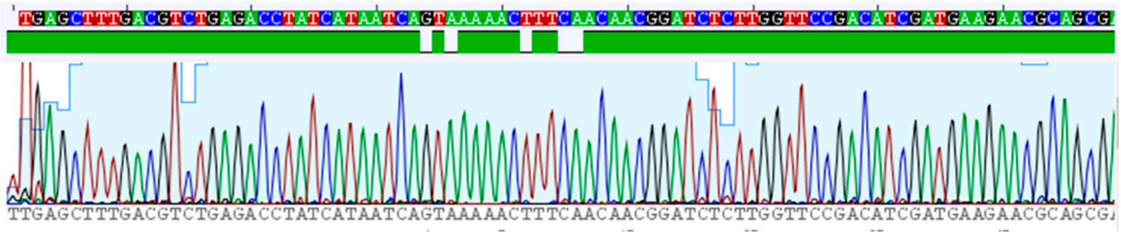

Sequences producing significant alignments:

Select: All None Selected:0

Alignments Download GenBank Graphics Distance tree of results

Description	Max score	Total score	Query cover	E value	Ident	Accession
Paracoccidioides brasiliensis isolate T16B1 internal transcribed spacer 1, partial sequence; 5.8S ribosomal RNA gene and internal transcribed	761	761	99%	0.0	100%	gi|386276209|JQ675762.1
Paracoccidioides brasiliensis internal transcribed spacer 1, partial sequence; 5.8S ribosomal RNA gene, complete sequence; and internal trans	761	761	99%	0.0	100%	gi|338843268|JF289270.1
Paracoccidioides brasiliensis small subunit rRNA, ITS1, 5.8S ribosomal RNA, ITS2, large subunit rRNA, partial and complete seqeunce, strain:	761	761	99%	0.0	100%	gi|147898339|AB304414.1
Paracoccidioides brasiliensis genes for 18S rRNA, ITS1, 5.8S rRNA, ITS2, 28S rRMA, partial and complete sequence	761	761	99%	0.0	100%	gi|110796947|AB035710.2

Figure S4. Sequence and blast of the ITS *loci* using the online tool Nucleotide Blast available at https://blast.ncbi.nlm.nih.gov.

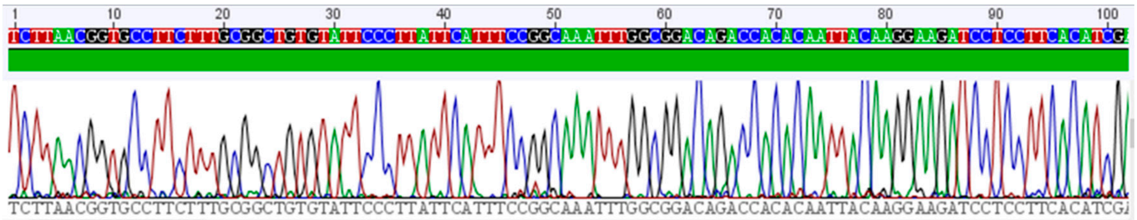

Sequences producing significant alignments:

Select: All None Selected:0

Alignments Download GenBank Graphics Distance tree of results

Description	Max score	Total score	Query cover	E value	Ident	Accession
Paracoccidioides brasiliensis strain B27 chitin synthase 2 (chs2) gene, promoter region and partial cds	1074	1074	100%	0.0	100%	gi|113967855|DQ640004.1
Paracoccidioides brasiliensis chs2 gene for chitin synthase, partial cds, strain: IFM 41622	1074	1074	100%	0.0	100%	gi|148277116|AB304538.1
Paracoccidioides brasiliensis strain B4 chitin synthase (chs2) gene, exons 2 through 4 and partial cds	1068	1068	100%	0.0	99%	gi|66934405|DQ004188.1
Paracoccidioides brasiliensis strain B3 chitin synthase (chs2) gene, exons 2 through 4 and partial cds	1068	1068	100%	0.0	99%	gi|66934403|DQ004187.1

Figure S5. Sequence and blast of the CHS2 gene using the online tool Nucleotide Blast available at https://blast.ncbi.nlm.nih.gov.

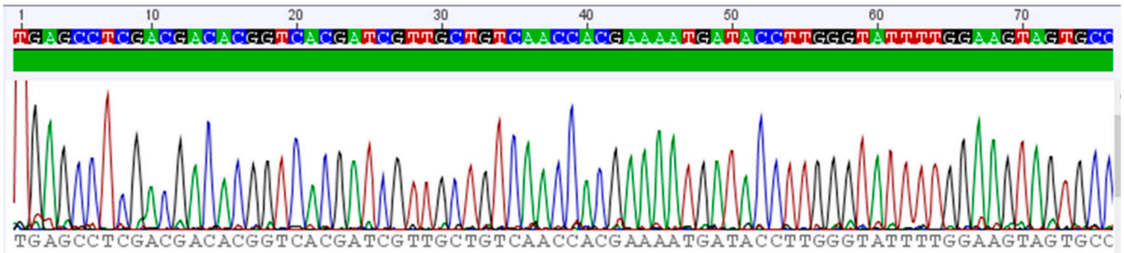

Sequences producing significant alignments:

Select: All None Selected:0

Alignments Download GenBank Graphics Distance tree of results

Description	Max score	Total score	Query cover	E value	Ident	Accession
Paracoccidioides brasiliensis strain B1 ADP-ribosylation factor (arf) gene, exons 2, 3 and partial cds	730	730	100%	0.0	100%	gi|66934127|DQ004049.1
Paracoccidioides brasiliensis ADP-ribosylation factor (Arf) gene, exons 2 and 3 and partial cds	725	725	100%	0.0	99%	gi|11992748|AY013311.1
Paracoccidioides brasiliensis strain A7 ADP-ribosylation factor (arf) gene, exons 2, 3 and partial cds	725	725	100%	0.0	99%	gi|66934245|DQ004108.1
Paracoccidioides brasiliensis strain B18 ADP-ribosylation factor (arf) gene, exons 2, 3 and partial cds	725	725	100%	0.0	99%	gi|66934161|DQ004066.1

Figure S6. Sequence and blast of the ARF gene using the online tool Nucleotide Blast available at https://blast.ncbi.nlm.nih.

Table S1. The GenBank accession numbers for the sequences used to construct our phylogenetic trees.

Sample Identifier	GenBank Accession Number (ITS)	GenBank Accession Number (CHS2 gene)	GenBank Accession Number (ARF gene)
FT1	OQ363050	OQ398644	OQ398607
FT2	OQ363108	OQ398645	OQ398608
FT3	OQ363109	OQ398646	OQ398609
FT4	OQ363110	OQ398647	OQ398610
FT5	OQ363111	OQ398648	OQ398611
FFPE_2	OQ363113	OQ398649	OQ398612
FFPE_3	OQ372105	OQ398650	OQ398613
FFPE_4	OQ363116	OQ398651	OQ398614
FFPE_5	OQ372091	OQ398652	OQ398615
FFPE_6	OQ363135	OQ398653	OQ398616
FFPE_9	OQ372094	OQ398654	OQ398617
FFPE_11	OQ372082	OQ398655	OQ398618
FFPE_14	OQ372090	OQ398656	OQ398619
FFPE_15	OQ372088	OQ398657	OQ398620
FFPE_16	OQ372085	OQ398658	OQ398621
FFPE_17	OQ372084	OQ398659	OQ398622
FFPE_18	OQ372093	OQ398660	OQ398623
FFPE_19	OQ372100	OQ398661	OQ398624
FFPE_20	OQ372092	OQ398662	OQ398625
FFPE_21	OQ372087	OQ398663	OQ398626
FFPE_22	OQ372102	OQ398664	OQ398627
FFPE_24	OQ363174	OQ398665	OQ398628
FFPE_25	OQ372097	OQ398666	OQ398629
FFPE_26	OQ372089	OQ398667	OQ398630
FFPE_27	OQ372083	OQ398668	OQ398631
FFPE_28	OQ372098	OQ398669	OQ398632
FFPE_29	OQ372086	OQ398670	OQ398633
FFPE_30	OQ372101	OQ398671	OQ398634
FFPE_31	OQ372107	OQ398672	OQ398635
FFPE_32	OQ372096	OQ398678	OQ398636
FFPE_33	OQ372108	OQ398674	OQ398637
FFPE_34	OQ372106	OQ398676	OQ398638
FFPE_35	OQ372103	OQ398677	OQ398639
FFPE_37	OQ372104	OQ398678	OQ398640
FFPE_38	OQ372109	OQ398679	OQ398641
FFPE_40	OQ372095	OQ398680	OQ398642
FFPE_42	OQ372099	OQ398675	OQ398643

Table S2. The GenBank accession numbers for the sequences used to compare with our isolates and construct the ARF and CHS2 phylogenetic tree.

Sample Identifier	GenBank Accession Number (CHS2 gene)	GenBank Accession Number (ARF gene)
B10 (S1 species)	DQ004193.1	DQ004058.1
B16(S1 species)	DQ004243.1	DQ004064.1
B7 (PS2 species)	DQ004179.1	DQ004055.1
B13 (PS2 species)	DQ004181.1	DQ004061.1
B26 (PS2 species)	DQ004183.1	DQ004113.1
C1 (PS3 species)	DQ004205.1	DQ004074.1
C10 (PS3 species)	DQ004214.1	DQ004083.1
C17 (PS3 species)	DQ004221.1	DQ004090.1
218 (*P. lutzii*)	EU870232.1	EU870333.1
717 (*P. lutzii*)	EU870230.1	EU870320.1
769 (*P. lutzii*)	EU870231.1	EU870332.1
